# DNA Replication Is an Integral Part of the Mouse Oocyte’s Reprogramming Machinery

**DOI:** 10.1371/journal.pone.0097199

**Published:** 2014-05-16

**Authors:** Bingyuan Wang, Martin J. Pfeiffer, Caroline Schwarzer, Marcos J. Araúzo-Bravo, Michele Boiani

**Affiliations:** 1 Max Planck Institute for Molecular Biomedicine, Münster, Germany; 2 Group of Computational Biology and Systems Biomedicine, Biodonostia Health Research Institute, San Sebastián, Spain; 3 IKERBASQUE, Basque Foundation for Science, Bilbao, Spain; The Babraham Institute, United Kingdom

## Abstract

Many of the structural and mechanistic requirements of oocyte-mediated nuclear reprogramming remain elusive. Previous accounts that transcriptional reprogramming of somatic nuclei in mouse zygotes may be complete in 24–36 hours, far more rapidly than in other reprogramming systems, raise the question of whether the mere exposure to the activated mouse ooplasm is sufficient to enact reprogramming in a nucleus. We therefore prevented DNA replication and cytokinesis, which ensue after nuclear transfer, in order to assess their requirement for transcriptional reprogramming of the key pluripotency genes *Oct4 (Pou5f1)* and *Nanog* in cloned mouse embryos. Using transcriptome and allele-specific analysis, we observed that hundreds of mRNAs, but not Oct4 and Nanog, became elevated in nucleus-transplanted oocytes without DNA replication. Progression through the first round of DNA replication was essential but not sufficient for transcriptional reprogramming of *Oct4* and *Nanog*, whereas cytokinesis and thereby cell-cell interactions were dispensable for transcriptional reprogramming. Responses similar to clones also were observed in embryos produced by fertilization *in vitro.* Our results link the occurrence of reprogramming to a previously unappreciated requirement of oocyte-mediated nuclear reprogramming, namely DNA replication. Nuclear transfer alone affords no immediate transition from a somatic to a pluripotent gene expression pattern unless DNA replication is also in place. This study is therefore a resource to appreciate that the quest for always faster reprogramming methods may collide with a limit that is dictated by the cell cycle.

## Introduction

When gently manipulated and properly cultured *in vitro*, most metaphase II (MII) mouse oocytes receiving a somatic cell nucleus transplant (SCNT) give rise to cloned embryos that recapitulate features of normal cleavage e.g. pluripotent gene expression albeit with a time delay [1]. Collectively these features are named ‘oocyte-mediated reprogramming’. This reprogramming machinery has been proposed to work astoundingly fast, with genes being reactivated within 24 hours after SCNT [2]. The components of the oocyte’s reprogramming machinery are known only in part. Maternal-effect factors [3], [4] make good candidates. Additional candidates are portrayed through a set of 28 proteins referred to as the ‘reprogrammome’ [5]. One would also like to link these candidate reprogramming factors to specific mechanisms. However, analyzing the reprogramming mechanism(s) is convoluted because multiple processes like DNA replication and cell divisions coincide with the reprogramming itself.

In particular, DNA replication temporarily disrupts the chromatin-nucleosome structure, providing a window of opportunity for oocytic reprogramming factors to gain access to key DNA sequences and to activate pluripotency genes that are silent in the somatic nucleus, such as *Oct4* (*Pou5f1*) and *Nanog*. In addition to being more accessible, key DNA sequences are also transiently hemimethylated during DNA replication i.e. carry reduced DNA methylation, which may facilitate derepression of silent somatic genes needed in the cloned embryo. *Oct4* is the best-studied gene of developmental pluri/totipotency. It encodes a POU-domain transcription factor that is essential for the survival of primordial germ cells and for the maintenance of a pluripotent state in inner cell mass (ICM) and embryonic stem (ES) cells [7], [8]. Along with OCT4, NANOG is another key transcriptional regulator expressed by pluripotent cells. Like OCT4, NANOG is required for the maintenance of pluripotency in mouse ICM and ES cells [9]. These two genes start to be expressed at the 4- to 8-cell stage of mouse development.

The requirement of DNA replication for reprogramming is mostly unexplored in oocytes, as opposed to reprogramming systems with high-throughput capacities, such as cell fusion and transcription factor (*Oct4, Sox2, c-Myc, Klf4*)-induced pluripotency [10]. After fusion between cells of different species e.g. human fibroblasts or B cells and mouse ES cells, the induction of human pluripotency genes e.g. *Oct4* and *Nanog* was detected in heterokaryons that were 94% negative for 5-Bromo-2′-deoxyuridine incorporation [11], but not in heterokaryons that were treated with the DNA polymerase inhibitor aphidicolin [12]. These contrasting results attest to the uncertainty that surrounds the role of DNA replication in reprogramming. When fusion was conducted between cells of the same species e.g. mouse F9 embryonal carcinoma (EC) cells and mouse *Oct4-GFP* transgenic neural stem cells (NSCs), the induction of the pluripotency marker OCT4-GFP was observed within 24 hours of fusion, suggesting that reprogramming occurred in one cell cycle and that a single round of DNA replication was sufficient [13]. In transcription factor-mediated reprogramming, increasing the rate of somatic cell division by inhibition of the p53/p21 pathway or by overexpression of *Lin28* accelerated the kinetics of induced pluripotent stem (iPS) cell formation [14]. However, Xu and colleagues reported that removing the pro-mitogenic *c-Myc* from the cocktail (*Oct4, Sox2, Klf4* but without *c-Myc*) or adding cell cycle inhibitors at the early stage of reprogramming increased the efficiency of iPS cell induction [15].

In immature *Xenopus* oocytes that were each transplanted with 100–200 human somatic nuclei, the human *Oct4* locus was activated without DNA replication, as shown by RT-PCR detection of human Oct4 mRNA 4 days after SCNT into the germinal vesicle [16]. An approach similar to *Xenopus* is infeasible in mammalian oocytes, which are small and fragile. Following SCNT of single nuclei in mouse oocytes, the embryonic cell cycle is often delayed, resulting in cloned embryos with fewer cells [1], perhaps due to delayed DNA replication. Interestingly, treatment of cloned embryos with trichostatin A or caffeine brings forward the initiation of DNA replication and the timing of the first cleavage [17]–[19].

In the present study, we sought to clarify whether the mere exposure of a cumulus cell nucleus to the activated mouse ooplasm, skipping DNA replication, allows for the transition from a somatic to a pluripotent gene expression pattern, as measured by transcriptional reprogramming of the pluripotency-associated genes *Oct4* and *Nanog*. Therefore we used allele-specific assays to distinguish between reprogrammed somatic mRNAs and preexisting oocytic mRNAs, in order to see how the somatic mRNAs would be expressed when the first or the second round of DNA replication is suppressed in SCNT embryos treated with aphidicolin (Aph). In the former case, the DNA of the transplanted nucleus would not be replicated at all, while in the latter case one single round of replication would be complete, allowing for opening of chromatin and access of oocytic factors. The use of Aph not only blocks DNA replication but also prevents cell division (cytokinesis) and therefore the establishment of a multicellular structure including cell-cell contacts. Thus, we tested for a possible role of cell-cell interactions in oocyte-mediated reprogramming using a different pharmacological treatment with cytochalasin B (CB); CB inhibits cytokinesis but not DNA synthesis. Our results show that nuclear transfer alone affords no immediate transition from a somatic to an Oct4- and Nanog-positive gene expression pattern, unless DNA replication is also in place in the mouse ooplasm. Unlike DNA replication, cell division and thereby cell-cell interactions are not necessary for transcriptional activation of these genes.

## Results

### Change of Gene Expression Pattern Occurs without DNA Replication after SCNT

To pursue the changes in transcription that result from SCNT and exchange of nucleus-cytoplasmic factors, the DNA replication that normally ensues after SCNT was inhibited using aphidicolin (Aph). Aph is a deoxyribonucleotide analogue competing with deoxycytidine triphosphate (dCTP) incorporation. It potently inhibits all replicative and most repair-associated DNA polymerases leading to cell cycle arrest at the G1/S phase border [20], [21].

**Figure 1:**
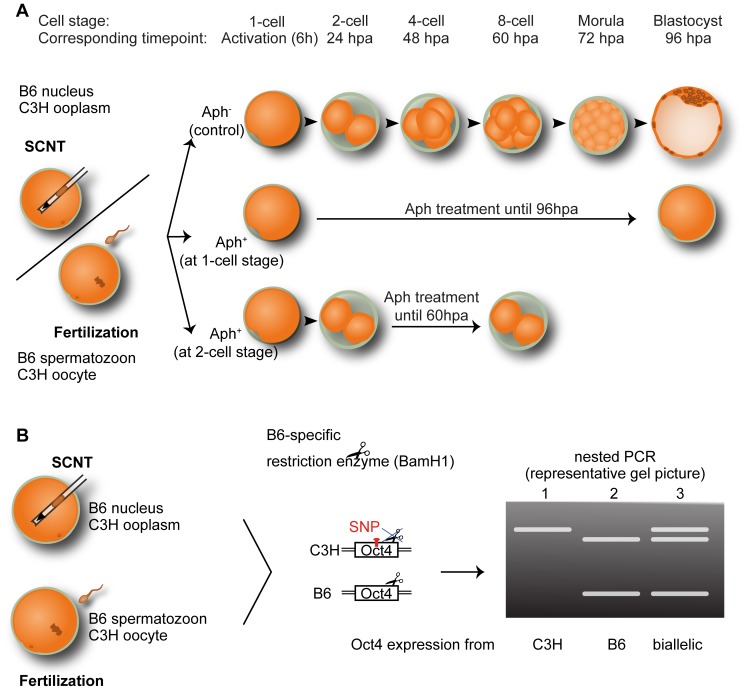
Experimental design for distinguishing the allelic origin of Oct4 mRNA in SCNT and fertilized embryos. (**A**) Enucleated C3H/HeN (for brevity, C3H) oocytes were transplanted with C57Bl/6J (for brevity, B6) cumulus cell nuclei to produce SCNT embryos, which were cultured in the presence of aphidicolin (Aph) or in normal medium, resulting in G1/S phase arrested pronuclear oocytes or in cleavage stages, respectively. Fertilized embryos were produced with intact C3H oocytes and B6 sperm and cultured the same way as the SCNT embryos. hpa: hours past activation. (**B**) Oct4 transcript from the C3H and B6 mouse strains can be distinguished by a restriction-enzyme-sensitive single nucleotide polymorphism (SNP). Oct4 transcript from C3H remains uncut (565 bp), while Oct4 transcript from B6 is cut (429 bp and 136 bp). If found positive for β-actin mRNA, embryos were then processed and analyzed individually.

Embryos were produced by SCNT or intracytoplasmic sperm injection (ICSI) (Figure 1A) using a scheme in which the oocyte and the nucleus donor’s mRNAs can be distinguished from each other by single nucleotide polymorphisms (SNPs) (Figure 1B). Enucleated C3H/HeN oocytes were transplanted with single nuclei from C57Bl/6J cumulus cells; intact C3H/HeN oocytes were injected with C57Bl/6J sperm. For ease of description we call these constructs C3HxB6. The resultant pronuclear oocytes (6 hours post activation, hpa) and 2-cell embryos (24 hpa) were both allocated randomly to two groups: one treated with 2 µg/ml Aph in the culture medium (Aph^+^) to suppress DNA replication, the other left untreated (Aph^−^) to allow DNA replication. Because Aph induces cell cycle arrest, Aph^+^ pronuclear oocytes remain at the 1-cell stage, Aph^+^2-cell embryos remain at the 2-cell stage, while Aph^−^ specimens can divide and progress in development (Figure 1A). Although the effect of Aph is considered to be reversible, reversibility and toxicity of Aph are dose- and time-dependent in mouse embryos [22]. The mouse embryos of this study could not resume with cleavage when 2 µg/ml Aph was washed off after 6-hour treatment.

**Figure 2:**
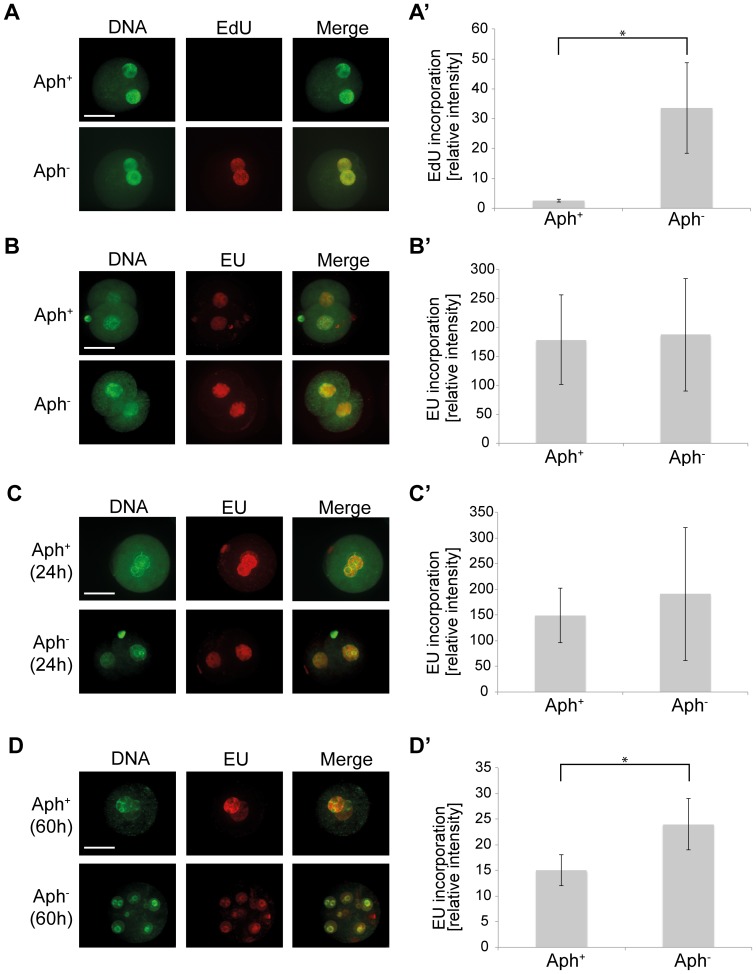
Aphidicolin effectively and selectively inhibits DNA synthesis in fertilized embryos. In mouse development the first round of DNA replication occurs at the pronuclear stage and the major wave of embryonic genome activation occurs at the 2-cell stage. While the deoxyribonucleotide analogue 5-ethynyl-2′-deoxyuridine (EdU) was incorporated into newly synthesized DNA of aphidicolin-negative (Aph^−^) pronuclear oocytes, no EdU incorporation was detected in Aph^+^ pronuclear oocytes that were treated with 2 µg/ml Aph for 6 hours (**A, A’**). When 2-cell embryos in G1 phase were cultured with the ribonucleotide analogue 5*-*ethynyl uridine (EU), EU was incorporated into newly synthesized mRNA regardless of the 6-hour Aph treatment (**B, B’**). When Aph treatment was protracted after 6 hours, EU incorporation was unchanged after 24 hours (**C, C’**) and was reduced after 60 hours (**D, D’**). Incorporation of EdU and EU was revealed using Click-iT imaging technology. Fluorescence images were taken on a Nikon TE2000 microscope fitted with an UltraView RS3 confocal module, and the fluorescence signals indicative of EdU and EU incorporation were quantified using ImageJ. Scale bar 40 µm. Statistical significance was calculated using t-test with p≤0.05.

The effectiveness and selectivity of Aph toward DNA synthesis was confirmed by analyzing the incorporation of nucleotide analogues into DNA and mRNA. Incorporation of 5-ethynyl-2′-deoxyuridine (EdU) and 5-ethynyl uridine (EU) into DNA and mRNA, respectively, was measured using Click-iT imaging technology. Treatment with Aph prevented EdU incorporation into DNA (Figure 2A,A’) but not EU incorporation into mRNA (Figure 2B,B’,C,C’,D,D’), consistent with the effects reported in sea urchin embryos [23]. Thus, mRNA transcription still occurs with suppressed DNA replication, albeit at a seemingly reduced level. Therefore, we asked if the global mRNA profile characteristic of normal development is also reproduced when DNA replication is suppressed, featuring the accumulation of mRNAs e.g. Oct4 and Nanog over time [24]. At 96 hpa mRNA was extracted from pools of Aph^+^ embryos (1 cell-arrested) that were sampled randomly, and from Aph^−^ embryos that were sampled regardless of the stage attained so as to respect the initial proportions of pronuclear oocytes that were competent vs. not competent for development. These mRNA samples were collected in triplicates from both SCNT and fertilized embryos, and were subjected to microarray analysis.

**Figure 3:**
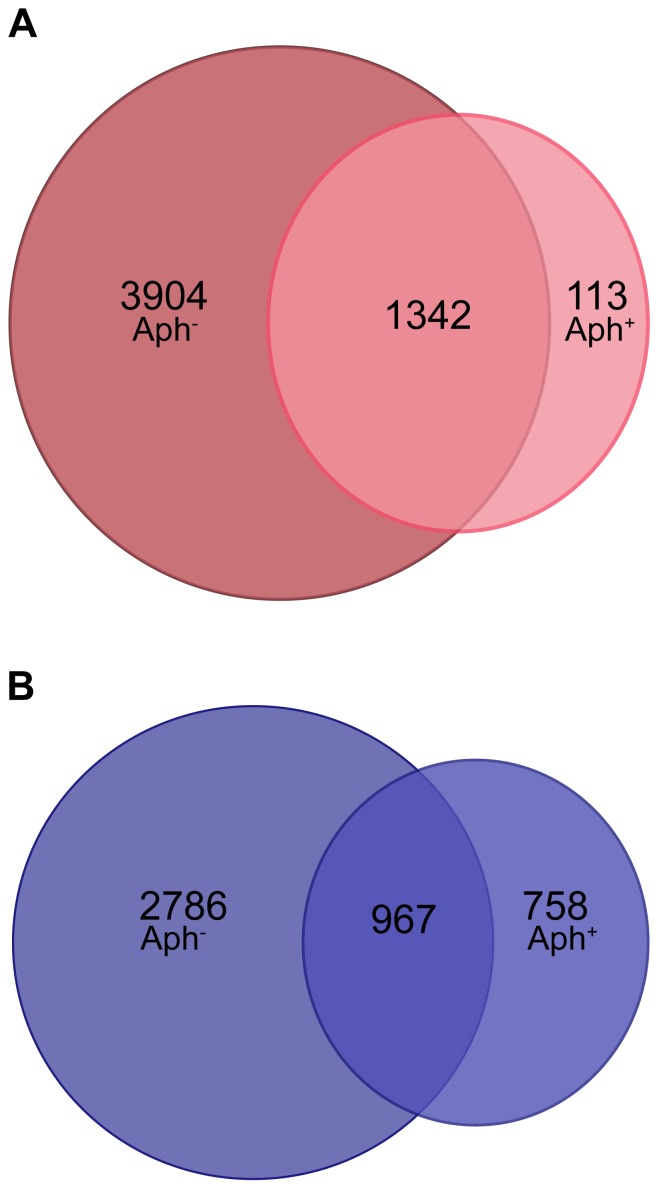
Venn diagram of transcripts elevated ≥2-fold in embryos treated or not treated with aphidicolin. Pronuclear oocytes were cultured in the presence of aphidicholin (Aph) from 6 to 96 hours post activation (hpa), and compared with sibling embryos that were not treated so as to appreciate how many mRNAs are upregulated regardless of the cell cycle progression. (**A**) Venn diagram showing mRNAs that accumulate after SCNT when the first round of embryonic DNA replication is suppressed (Aph^+^) in comparison to mRNAs that accumulate when the embryo can cycle normally (Aph^−^). (**B**) Venn diagram showing mRNAs that accumulate after fertilization when the first round of embryonic DNA replication is suppressed (Aph^+^) in comparison to mRNAs that accumulate when the embryo can cycle normally (Aph^−^). In both SCNT and fertilization, mRNAs were considered whose abundance increased at least 2-fold compared to MII oocytes.

The results of the transcriptome analysis indicate that a subset (≈25%) of embryonic genes can still be upregulated when DNA replication is suppressed from the first round (Figure 3A,B). We used the criterion of ≥2-fold abundance increase relative to MII oocytes - the common starting material of SCNT and fertilization. For SCNT, 5246 mRNAs increased ≥2-fold in the Aph^−^ group (with DNA replication); of these mRNAs, 1342 (25.6%) also increased in the Aph^+^ group (without DNA replication) (Figure 3A). For fertilization, 3753 mRNAs increased ≥2-fold in the Aph^−^ group; of these mRNAs 967 (25.8%) also increased in the Aph^+^ group (Figure 3B; these differently expressed genes are listed in Table S1). We asked if the mRNAs that increased ≥2-fold during suppressed DNA replication had any special functional annotation. Gene ontology (GO) terms of the GO ‘biological process’ featured highly significant enrichment in the terms ‘mRNA processing’ and ‘translation’, after both SCNT and fertilization (FDR, q<0.01 with Benjamini-Hochberg correction for multiple comparisons, Table S1).

### First Round of DNA Synthesis is Necessary for Transcriptional Activation of Somatic and Sperm *Oct4* Gene

**Figure 4:**
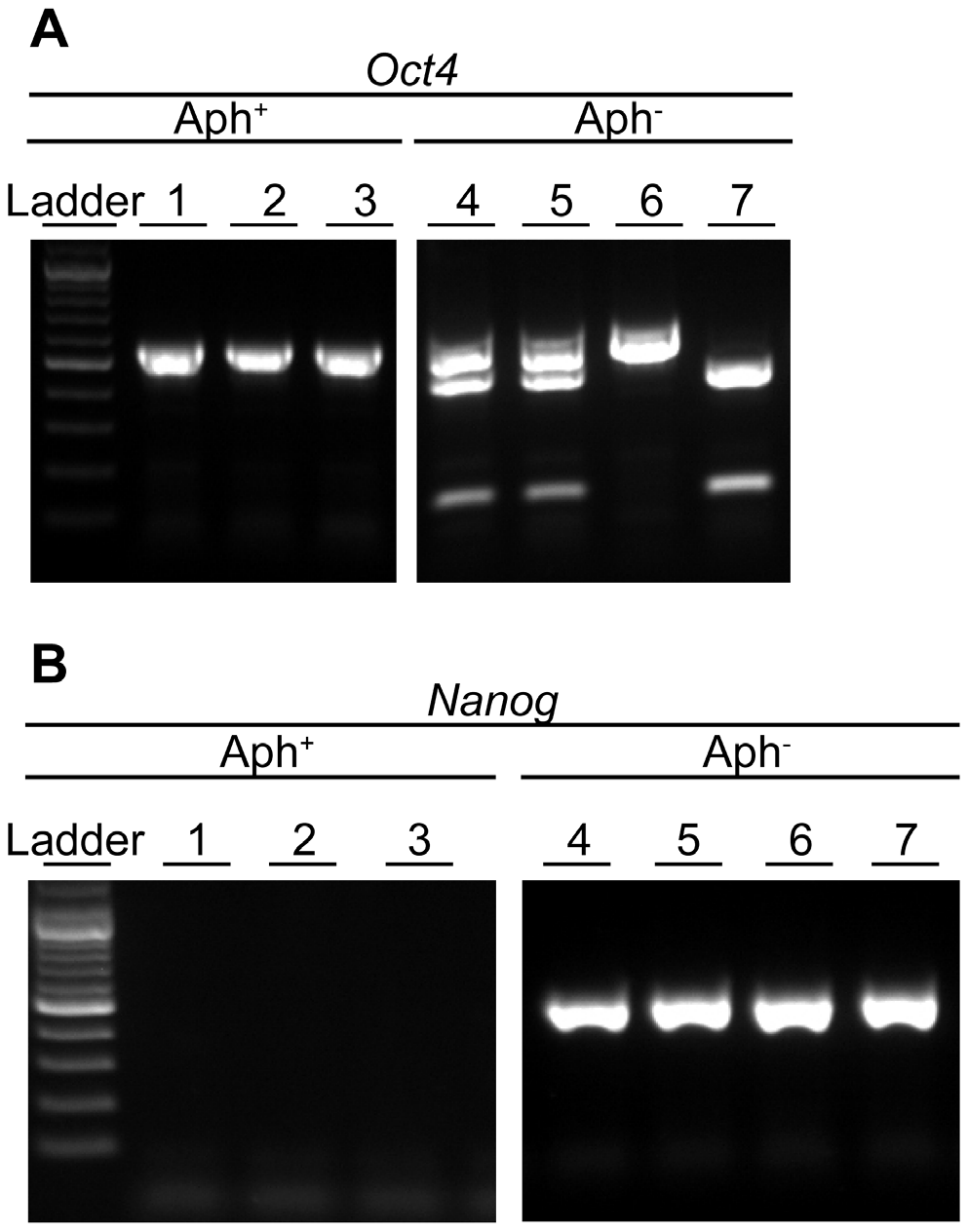
Representative images of individual embryos analyzed by allele-specific PCR for Oct4 and Nanog mRNA. All of the embryos subjected to Oct4 (**A**) and Nanog (**B**) analysis were previously found positive for β-actin mRNA. Gel pictures show 7 embryos analyzed for Oct4 and 7 embryos analyzed for Nanog. (**A**) Oct4 transcripts from 3 Aph^+^ embryos are only maternal (C3H/HeN amplicon, single band uncut), whereas Oct4 transcript from 4 Aph^−^ embryos are either maternal (no reprogramming) or somatic (C57Bl/6J reprogrammed, cut) or paternal (C57Bl/6J sperm, cut). (**B**) Aph^+^ embryos have no Nanog transcript whereas Aph^−^ embryos have Nanog transcript from either the C3H/HeN or the C57Bl/6J allele.

The previous analysis was performed on samples consisting of multiple oocytes; although this is a necessary strategy for global characterization, it obscures the heterogeneity of nucleus-transplanted oocytes. To analyze *Oct4* gene expression in individual nucleus-transplanted oocytes, mRNA was isolated from Aph^+^ and Aph^−^ embryos that were sampled at 24, 48, 60, and 72–96 hpa, which would correspond to the 2-cell, 4-cell, 8-cell and morula/blastocyst stage (Figure 1A). Experiments were repeated 6–7 times and embryos were processed individually. After mRNA extraction, reverse transcription and PCR, samples were analyzed for the housekeeping mRNA β-actin and processed further if positive. The cDNA of C3H/HeN Oct4 mRNA (oocytic) cannot be cut by the restriction enzyme BamH1, whereas the cDNA of C57Bl/6J Oct4 mRNA (somatic or sperm) can be cut (Figure 4A), allowing to discriminate if its origin was somatic (after SCNT)/paternal (after ICSI) or oocytic.

**Figure 5:**
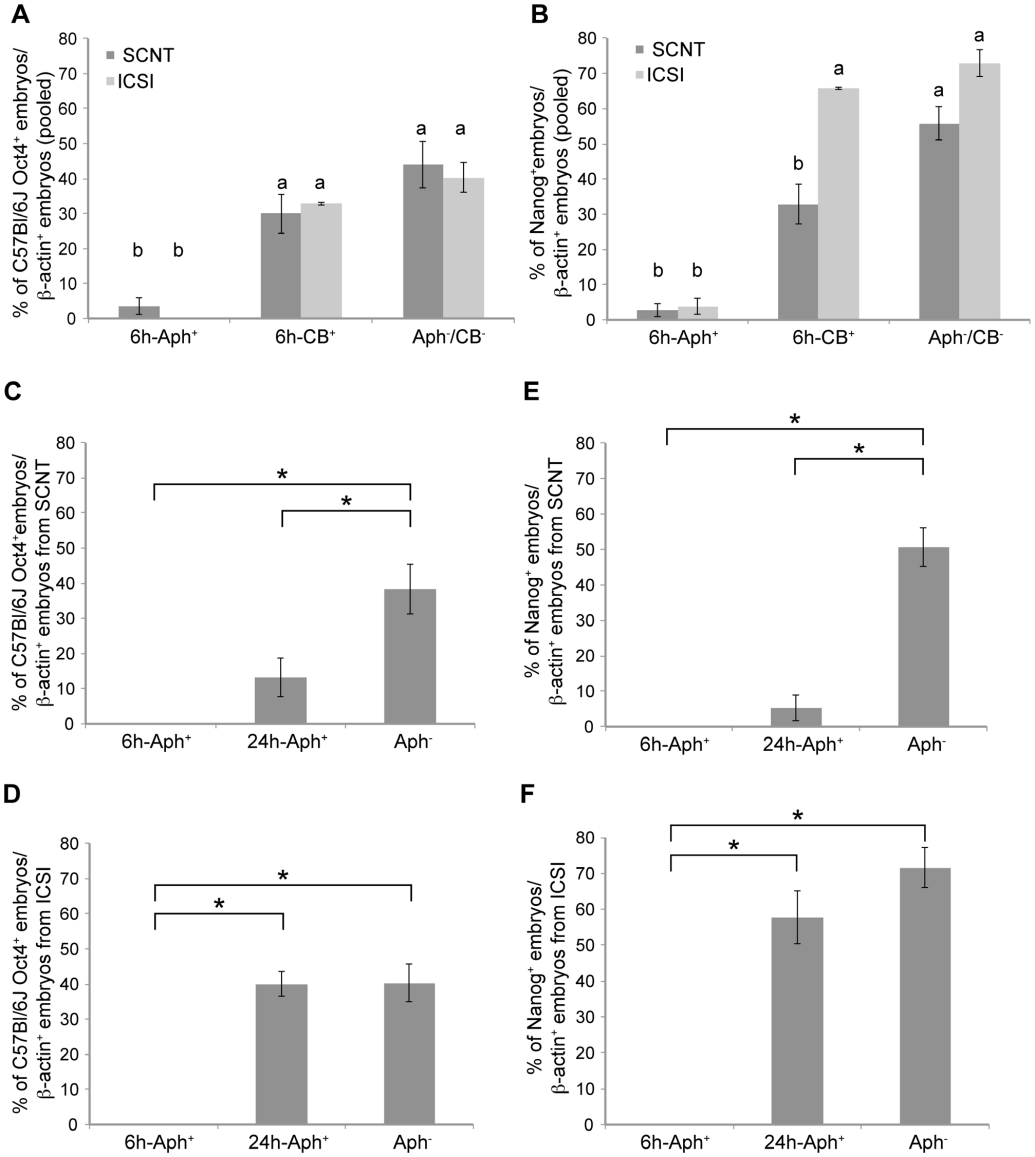
Results of individual embryos analyzed by allele-specific PCR for Oct4 and Nanog mRNA. (**A**) Pooled frequencies of Oct4 and (**B**) Nanog mRNA expression in SCNT or ICSI embryos after treatment with Aph or CB from 6 hpa. (**C**) Frequencies of Oct4 mRNA expression in SCNT embryos after treatment with Aph or CB from 6 or 24 hpa. (**D**) Frequencies of Oct4 mRNA expression in ICSI embryos after treatment with Aph or CB from 6 or 24 hpa. (**E**) Frequencies of Nanog mRNA expression in SCNT embryos after treatment with Aph or CB from 6 or 24 hpa. (**F**) Frequencies of Nanog mRNA expression in ICSI embryos after treatment with Aph or CB from 6 or 24 hpa. All of the abovementioned embryos tested positive for β-actin mRNA. Statistical significance was calculated using chi-square test with p≤0.05.

We compared the frequencies of Oct4 mRNA expression in Aph^+^ and Aph^−^ specimens (β-actin mRNA positive) using the chi-square test. The results of RT-PCR analysis are summarized in Table 1. At 24 hpa C57Bl/6J Oct4 mRNA was not detected in any of the C3HxB6 embryos analyzed (data not shown). At 48, 60 and 72–96 hpa, 7%, 0% and 4% of the Aph^+^ SCNT embryos expressed the C57Bl/6J Oct4 mRNA, respectively, as compared to 13%, 36% and 64% of Aph^−^ counterparts (see Table 1 for pairwise comparisons; pooled, p = 4.04E-07; Figure 5A). Analogous to SCNT, after ICSI Aph^+^ embryos presented reduced frequencies of C57Bl/6J Oct4 mRNA, as compared to Aph^−^ embryos (see Table 1 for pairwise comparisons; pooled, p = 9.17E-11; Figure 5A). Notably, a substantial proportion of SCNT and ICSI embryos positive for β-actin mRNA were negative for Oct4 mRNA, whether C3H/HeN or C57Bl/6J (Table 1). By contrast, 100% (12/12) and 75% (9/12) of oocytes and blastocysts recovered *in vivo* and processed immediately for analysis were positive for Oct4 mRNA (data not shown).

**Table 1:** PCR detection of Oct4 mRNA in SCNT and ICSI embryos (C3HxB6).

	No. embryos B6 Oct4^+^/total Oct4^+^/β-actin^+^ (% B6 Oct4^+^/β-actin^+^)		
Groups	Aph^−^	Aph^+^	CB^+^
**48 hpa SCNT (treated at 6 hpa)**	2/8/15 (13)^a^	1/8/14 (7)^a^	0/3/10 (0)^a^
**48 hpa ICSI (treated at 6 hpa)**	2/8/14 (14)^a^	0/7/14 (0)^a^	4/6/8 (50)^a^
**60 hpa SCNT (treated at 6 hpa)**	5/5/14 (36)^a^	0/5/18 (0)^b^	8/9/34 (24)^a^
**60 hpa SCNT (treated at 24 hpa)**	13/13/33 (39)^a^	5/6/38 (13)^b^	n.p.
**60 hpa ICSI (treated at 6 hpa)**	16/21/50 (32)^a^	0/6/24 (0)^b^	13/25/69 (19)^a^
**60 hpa ICSI (treated at 24 hpa)**	11/14/17 (65)^a^	18/28/45 (40)^a^	n.p.
**72–96 hpa SCNT (treated at 6 hpa)**	18/18/28 (64)^a^	1/8/25 (4)^b^	12/12/23 (52)^a^
**72–96 hpa ICSI (treated at 6 hpa)**	34/40/65 (52)^a^	0/2/40 (0)^b^	27/36/57 (47)^a^
**Pooled SCNT (treated at 6 hpa)**	25/31/57 (44)^a^	2/21/57 (4)^b^	20/24/67 (30)^a^
**Pooled ICSI (treated at 6 hpa)**	52/69/129 (40)^a^	0/15/78 (0)^b^	44/77/134 (33)^a^

Pronuclear stage oocytes at 6 hours post activation (hpa) following SCNT were cultured in 2 µg/mL aphidicolin (aph) or in 5 µg/mL cytochalasin B (CB) until 48, 60 and 72–96 hpa to test the requirement of the first round of DNA replication and cell division for reprogramming. As controls, pronuclear stage oocytes were treated the same way after ICSI, or were cultured in normal medium and allowed to cleave to 2-cell, 4-cell and morula-blastocyst stage. To test the requirement of the second round of DNA replication and cell division for reprogramming, 2-cell embryos at 24 hpa were cultured in 2 µg/mL aph or in 5 µg/mL CB until 60 hpa and were compared with time-matched 8-cell embryos. As controls, 2-cell embryos were treated the same way after ICSI. For each treatment and type of embryo, results are given as ratio of frequencies, as follows: embryos expressing C57Bl/6J Oct4 mRNA/embryos expressing Oct4 mRNA/embryos positive for β-actin mRNA. Statistical analysis was conducted on the ratio C57Bl/6J Oct4/β-actin mRNA using chi-square test and applying the Bonferroni correction for multiple comparisons.

a,b : different superscripts within same row indicate significant difference (chi-square test).

n.p.: not performed.

total Oct4: Oct4 from maternal, paternal/somatic, or bi-allelic origins.

**Figure 6:**
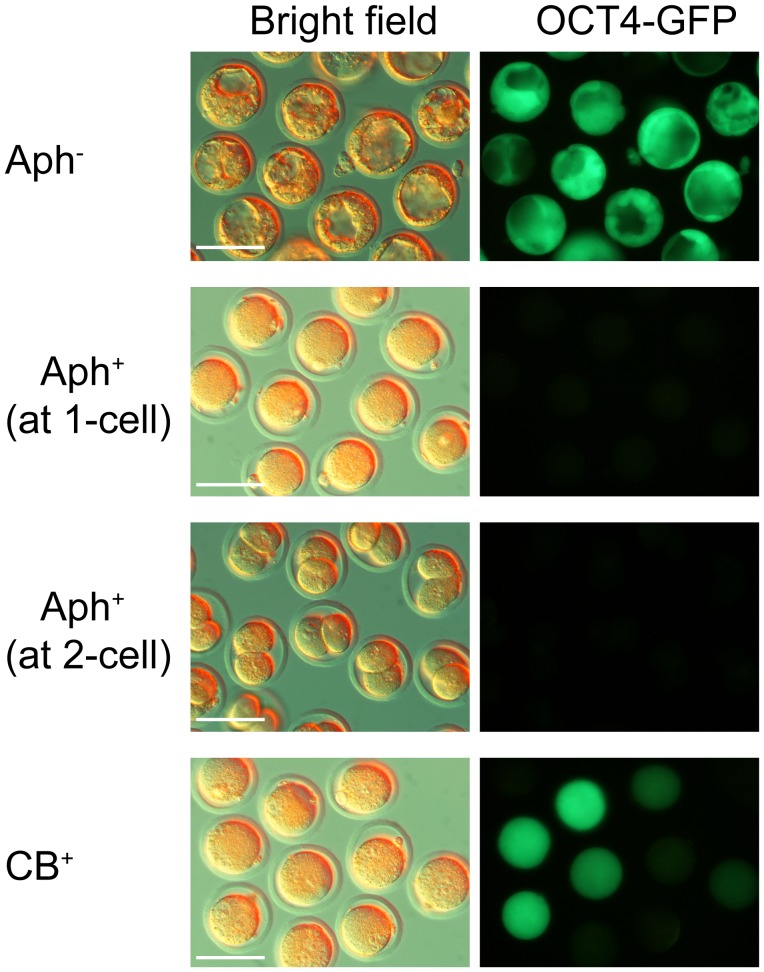
Representative images of Aphidicolin (Aph) or cytochalasin B (CB) treated embryos derived from *Oct4-GFP* nuclei. Aph^−^: control embryos not treated with Aph cleave as expected and express OCT4-GFP. Aph^+^: embryos treated with 2 µg/mL Aph at the 1- or 2-cell stage, which blocks DNA replication and thereby cell division (cytokinesis), fail to express OCT4-GFP. CB: treatment with 5 µg/mL CB prevents cytokinesis without affecting DNA synthesis and OCT4-GFP expression. Scale bar 100 µm.

An *Oct4-GFP* transgene (OG2 mice) gave us the opportunity to reach beyond mRNA and see if the green fluorescent protein that reflects the *Oct4* promoter activity would be produced after suppression of the first round of DNA replication. Accordingly, C3H/HeN oocytes received an *Oct4-GFP* cumulus cell nucleus or were fertilized with *Oct4-GFP* sperm from OG2 mice. Upon regular cleavage, these oocytes turned into green-fluorescent blastocysts (Figure 6); however, no specimen in the Aph^+^ group was positive for GFP, neither after SCNT nor after ICSI (Table 2). The bright field images document the morphology of embryos cultured from 6 to 96 hpa in the presence of Aph (Figure 6).

**Table 2:** OCT4-GFP expression in SCNT and ICSI embryos (C3HxOG2).

	GFP^+^/total (%)		
Groups	Aph^−^	Aph^+^	CB^+^
**96 hpa SCNT (treated at 6 hpa)**	59/65 (90.8)^a^	0/52 (0)^b^	28/75 (37.3)^b^
**96 hpa SCNT (treated at 24 hpa)**	59/65 (90.8)^a^	0/211 (0)^b^	8/13 (61.5)^b^
**96 hpa ICSI (treated at 6 hpa)**	36/36 (100)^a^	0/27 (0)^b^	45/54 (83.3)^b^
**96 hpa ICSI (treated at 24 hpa)**	36/36 (100)^a^	n.p.	28/28 (100)^a^

To test the requirement of first round DNA replication and cell division for reprogramming, pronuclear oocytes at 6 hours post activation (hpa) were cultured in 2 µg/mL aphidicolin (aph) or in 5 µg/mL cytochalasin B (CB) until 96 hpa. In parallel, pronuclear oocytes were allowed to cleave and reach the blastocyst stage. To test the requirement of second round DNA replication and cell division for reprogramming, 2-cell embryos at 24 hpa were cultured in 2 µg/mL aph or in 5 µg/mL CB until 96 hpa and were compared with time-matched blastocyst-stage embryos. For each treatment and type of embryo, results are given as frequencies of embryos expressing GFP/embryos imaged. These frequencies, given as percent, were analyzed using chi-square test and applying the Bonferroni correction for multiple comparisons.

a,b : different superscripts within same row indicate significant difference (chi-square test).

n.p.: not performed.

We then tested the role of the second round of DNA replication in transcriptional reprogramming of *Oct4*. C3HxB6 embryos were subjected to Aph treatment as described except that they were treated at 24 hpa, just after they had reached the 2-cell stage (Figure 1A). In this case, one cell division and one single round of replication would have completed, allowing for opening of chromatin and access of reprogramming factors to the *Oct4* locus. At 60 hpa the Aph^+^ embryos (2-cell arrested) and the Aph^−^ embryos (progressed further) were examined individually for the presence of β-actin and Oct4 mRNA. Compared to the permanent suppression of DNA replication, suppression from the 2-cell stage onward allowed for more SCNT embryos to express the C57Bl/6J Oct4 mRNA (rising from 0% to 13% of the β-actin mRNA-positive embryos; chi-square test, p = 0.11; Table 1; Figure 5C). This increase lags behind the increase observed in ICSI embryos (from 0% to 40% of the β-actin mRNA-positive embryos; chi-square test, p = 0.0003; Table 1; Figure 5D). When C3H/HeN oocytes received an *Oct4-GFP* cumulus cell nucleus or were fertilized with *Oct4-GFP* sperm from OG2 mice, no specimen was positive for GFP fluorescence in the Aph^+^ group, whether SCNT or ICSI, in contrast to the Aph^−^ group (Table 2; Figure 6). The bright field images document the morphology of embryos cultured from 24 to 96 hpa in the presence of Aph (Figure 6).

Taken together, these data show that the first round of DNA synthesis is necessary for the transcriptional reprogramming of *Oct4* in SCNT embryos. However, the requirement for DNA replication is not unique to SCNT embryos, since *Oct4* expression from sperm alleles is also prevented when the first round of DNA synthesis is suppressed after fertilization. Failure to detect any Oct4 mRNA in a proportion of the ICSI embryos positive for β-actin mRNA attests to the detrimental effect of micromanipulation (including *in vitro* culture) on gene expression, regardless of somatic nuclear reprogramming.

### Insufficiency of the First Round of DNA Synthesis for Transcriptional Activation of Somatic and Sperm *Nanog* Gene

Like OCT4, NANOG is a key reprogramming factor and marker of pluripotency; unlike OCT4, NANOG is not present as maternal product in MII oocytes [25]. Therefore, the appearance of Nanog mRNA after SCNT is *per se* evidence that transcriptional reprogramming has occurred at this locus, waiving the need for allelic discrimination analysis (Figure 4B).

We compared frequencies of Nanog mRNA expression in Aph^+^ specimens with Aph^−^ specimens using the chi-square test. The results of RT-PCR analysis are summarized in Table 3. After SCNT, Nanog mRNA was not detected in any of the C3HxB6 embryos at 24 hpa (data not shown). At 48, 60 and 72–96 hpa, 6%, 0% and 4% of the Aph^+^ SCNT embryos express Nanog mRNA, respectively, as compared to 30%, 63%, 59% of Aph^−^ counterparts (see Table 3 for pairwise comparisons; pooled, p = 1.85E-13; Figure 5B). Analogous to SCNT, Aph treatment also reduced the frequency of Nanog mRNA expression after ICSI, as compared to Aph^−^ embryos (see Table 3 for pairwise comparisons; pooled, p = 5.24E-22; Figure 5B). Irrespective of SCNT or ICSI, frequencies of Nanog mRNA expression in Aph^−^ embryos (positive for β-actin mRNA) never reached 100% (Table 3). By contrast, 100% (12/12) of blastocysts recovered *in vivo* and processed immediately for analysis were positive for Nanog mRNA (data not shown).

**Table 3:** PCR detection of Nanog mRNA in SCNT and ICSI embryos (C3HxB6).

	No. embryos Nanog^+^/β-actin^+^ (%)		
Groups	Aph^−^	Aph^+^	CB^+^
**48 hpa SCNT (treated at 6 hpa)**	6/20 (30)^a^	1/17 (6)^a^	1/10 (10)^a^
**48 hpa ICSI (treated at 6 hpa)**	9/14 (64)^a^	1/14 (7)^b^	7/8 (88)^a^
**60 hpa SCNT (treated at 6 hpa)**	34/54 (63)^a^	0/27 (0)^b^	12/34 (35)^b^
**60 hpa SCNT (treated at 24 hpa)**	10/33 (30)^a^	2/38 (5)^b^	n.p.
**60 hpa ICSI (treated at 6 hpa)**	32/50 (64)^a^	0/24 (0)^b^	38/69 (55)^a^
**60 hpa ICSI (treated at 24 hpa)**	16/17 (94)^a^	26/45 (58)^b^	n.p.
**72–96 hpa SCNT (treated at 6 hpa)**	23/39 (59)^a^	1/28 (4)^b^	9/23 (39)^a^
**72–96 hpa ICSI (treated at 6 hpa)**	53/65 (82)^a^	2/40 (5)^b^	43/57 (75)^a^
**Pooled SCNT (treated at 6 hpa)**	63/113 (56)^a^	2/72 (3)^b^	22/67 (33)^b^
**Pooled ICSI (treated at 6 hpa)**	94/129 (73)^a^	3/78 (4)^b^	88/134 (66)^a^

Pronuclear oocytes at 6 hours post activation (hpa) following SCNT were cultured in 2 µg/mL aphidicolin (aph) or in 5 µg/mL cytochalasin B (CB) until 48, 60 and 72–96 hpa to test the requirement of the first round of DNA replication and cell division for reprogramming. As controls, pronuclear oocytes were treated the same way after fertilization, or were cultured in normal medium and allowed to cleave to 2-cell, 4-cell and morula-blastocyst stage. To test the requirement of the second round of DNA replication and cell division for reprogramming, 2-cell embryos at 24 hpa were cultured in 2 µg/mL aph or in 5 µg/mL CB until 60 hpa and were compared with time-matched 8-cell embryos. As controls, 2-cell embryos were treated the same way after fertilization. For each treatment and type of embryo, results are given as ratio of frequencies, as follows: embryos expressing Nanog mRNA/embryos positive for β-actin mRNA. Statistical analysis is conducted on the ratio Nanog/β-actin mRNA using chi-square test and applying the Bonferroni correction for multiple comparisons.

a,b : different superscripts within same row indicate significant difference (chi-square test).

n.p.: not performed.

We then tested the role of the second round of DNA replication in transcriptional reprogramming of *Nanog*. C3HxB6 embryos were subjected to Aph treatment at the 2-cell stage and examined individually for presence of Nanog mRNA at 60 hpa, when Nanog mRNA is detected in the majority of untreated counterparts. Compared to the permanent suppression of DNA replication, the proportion of SCNT embryos positive for Nanog mRNA did not increase when suppression took place from the 2-cell stage onward (0% vs. 5% of the β-actin mRNA-positive embryos, chi-square test, p = 0.23; Table 3; Figure 5E). ICSI embryos scored a marked increase of positives for Nanog mRNA (from 0% to 58% of the β-actin mRNA-positive embryos; chi-square test, p = 2.39E-06; Table 3; Figure 5F).

Taken together, these data show that the first round of DNA synthesis is not sufficient for the transcriptional reprogramming of *Nanog* in SCNT embryos. Furthermore, this observation is not unique to SCNT embryos, since *Nanog* expression is also prevented when the first round of DNA synthesis is suppressed after fertilization. Failure to detect Nanog mRNA in 44% of the SCNT and 27% of the fertilized morulae/blastocysts (positive for β-actin mRNA) suggests that preimplantation development unfolded in the absence of NANOG, which unlike OCT4, is not supplied to the embryo via the oocyte.

### Cell Division is Dispensable for Transcriptional Activation of *Oct4* and *Nanog* Genes in Cloned and Fertilized Mouse Embryos

Because Aph^+^ pronuclear-stage C3HxB6 embryos arrest at G1/S phase and do not progress in their cell cycle, cell-cell interactions that would normally arise during development and that might play a role in reprogramming also cannot form. To test if the results obtained can be accounted for by missing cell-cell interactions, we performed a drug treatment with 5 µg/mL cytochalasin B (CB), which prevents cell division (cytokinesis) without affecting DNA synthesis. As a result, the embryo remained at the 1-cell stage but became more and more polyploid [26]. The pronuclear-stage embryos were treated at the same time when we would have performed the Aph treatment (6 hpa), and were analyzed at 48, 60 and 72–96 hpa (Table 1) for frequencies of gene expression using the chi-square test. Among the SCNT embryos, pooled frequencies of somatic Oct4 mRNA expression were not significantly different in the CB^+^ (30%) and CB^−^ (Aph^−^, 44%) groups (chi-square test, p = 0.11)(Figure 5A). Among the fertilized embryos, pooled frequencies of sperm’s Oct4 mRNA expression were similar in the CB^+^ (33%) and CB^−^ (40%) groups (chi-square test, p = 0.21)(Figure 5A). When cumulus cells and sperm of *Oct4-GFP* mice were used, a substantial share of derivative embryos were positive for GFP fluorescence in the CB^+^ group, albeit with lower frequencies (37% and 83%, respectively; Table 2) or lower fluorescence intensity (Figure 6) than in the untreated group.

In the case of *Nanog* (Table 3), frequencies of mRNA detection were significantly different in the CB^+^ and CB^−^ groups after SCNT (33% vs 56%, chi-square test, p = 0.003) but were similar after fertilization (66% vs 73%, chi-square test, p = 0.21)(Figure 5B). These results show that cell-cell interactions are not required to activate the *Oct4* and *Nanog* loci, whether somatic or gametic; however, they may influence the penetrance or completeness of their activation after SCNT (Table 1, 3).

## Discussion

The central finding of our study is that nuclear transfer alone affords no immediate transition from a somatic to an Oct4- and Nanog-positive gene expression pattern, unless DNA replication is also in place within the mouse ooplasm. Without DNA replication from the first cell cycle, several mRNAs are elevated, but not Oct4 and Nanog, which are essential players of reprogramming in transcription factor-induced pluripotency. Allowing only the first round of embryonic DNA replication enables the transcriptional elevation of *Oct4* and to a lesser extent *Nanog*. Unlike DNA replication, cell division and thereby cell-cell interactions are not necessary for transcriptional activation of these genes. Similar effects were also observed in fertilized embryos.

As measured by microarray analysis conducted on pools of embryos, suppression of embryonic DNA replication from the first round using aphidicolin allows ≥2-fold upregulation of a subset (≈25%) of embryonic mRNAs that do not include Oct4 and Nanog. This observation was not unexpected. On the one hand, cell fusion studies revealed that certain genes that are silenced “in cis” may not be reprogrammed independent of DNA replication, as opposed to genes that are regulated “in trans” by trans-acting factors [27]. On the other hand, microarray analysis suffers from the averaging effect of this type of analysis conducted on pools of cells, whereby the slower accumulation of mRNAs transcribed from somatic loci in cloned mouse embryos [28], [29] makes it even less likely that these mRNAs will cross a 2-fold threshold. Therefore, we examined individual embryos for mRNAs of pluripotency genes *Oct4* and *Nanog* using a nested PCR assay.

The proportion of SCNT embryos expressing *Nanog* and somatic (C57Bl/6J) *Oct4* was reduced to almost nil when DNA replication was suppressed from the first round. A similar effect was observed in fertilized embryos. At variance with our observation, Byrne and colleagues reported on the transcription of the human and mouse somatic *Oct4* loci after cross-species SCNT into the *Xenopus* germinal vesicle (GV), which is a non-permissive environment for DNA replication [16]. We think our results and those of Byrne and colleagues can be reconciled with each other. We note that in Byrne and colleagues’ experimental setting, 100–200 nuclei were transplanted per GV, whereby the activation of *Oct4* in only a minority of the transplanted nuclei would have led to the interpretation that DNA replication is not a requirement for reprogramming. If Byrne and colleagues had examined reprogramming events i.e. nuclei individually, then their interpretation might have been similar to ours. Our mouse data, which are based on single transplanted nuclei, show that, when DNA replication was suppressed from the first round, transcriptional reprogramming only occurred in very rare exceptions.

In contrast to the total block of DNA replication from the first round, blocking DNA replication from the second round proved more permissive (albeit far from completeness) for the transcriptional activation of the somatic *Oct4* locus, while the somatic *Nanog* locus remained almost completely silent after SCNT. In regard to *Nanog*, we speculate that more requirements have to be fulfilled in order for it to be transcriptionally reprogrammed, as compared to *Oct4*. For example, *Nanog* is normally expressed in the embryo later than *Oct4*. In addition, *Nanog* gene activation cannot benefit from pre-existing (maternal) NANOG, which is not present in mature mouse oocytes, in contrast to maternal OCT4, which is already present in the C3H/H3N oocyte and might create an auto-feedback loop on the C57Bl/6J *Oct4* locus. Specifically, the relevant OCT4 would have to be OCT4B, since genetic ablation of *Oct4A* in oocytes is still permissive for somatic *Oct4-GFP* transgene induction after SCNT [30], [31].

As we sought to reach beyond the Oct4 mRNA by checking the appearance of OCT4 protein in SCNT oocytes lacking DNA replication, we faced the challenge of distinguishing between oocytic (maternal) and *de novo* synthesized OCT4 protein; mRNAs can be distinguished (by SNP) but proteins cannot, unless the SNP leads to non-synonymous codon change and this altered amino acid is detectable by an antibody. Therefore, we took advantage of the abovementioned *Oct4* promoter-driven GFP transgene delivered by the somatic nucleus or by sperm. This *Oct4-GFP* transgene has been used in many reprogramming studies to date, on the assumption that it reflects the expression of the endogenous *Oct4* [13], [32], [33]. Since OCT4-GFP fluorescence was previously observed 36 hours after SCNT [2], we were astonished to see no green fluorescence, neither in SCNT nor in fertilized embryos that skipped the first or second round of DNA replication and were aged 60 hpa. Counterparts treated with CB instead of Aph were green-fluorescent. It remains to be determined if the lack of OCT4-GFP fluorescence was an off-target effect of Aph on e.g. mRNA translation [26], or an indication that transcriptional reprogramming does not always predict actuation of reprogramming downstream of mRNA. It should also be noted that the *Oct4-GFP* transgene used in this study differs from the endogenous *Oct4* because it lacks the proximal enhancer, which is dispensable for transgene expression during normal cleavage but may adopt yet unknown secondary functions when DNA replication is suppressed.

Aph does not just block DNA synthesis. The drug also precludes cell-cell interactions because it arrests cell cycle progression at the G1/S border. Such interactions may be necessary to shape the pattern of embryonic gene expression [34]. Thus we assessed the effect of missing cell-cell interactions on reprogramming using the drug cytochalasin B (CB), which disrupts the actin cytoskeleton. As reported, treatment of mouse embryos with CB resulted in polyploid ooplasms and patterns of protein synthesis that change stage-specifically, as in control embryos through cleavage [26]. In our experiment, the treatment with CB to prevent cleavage enabled similar (p>0.05) frequencies of *Oct4* and *Nanog* allele reactivation compared to the untreated control. In *Xenopus* oocytes, nuclear actin has a role in nuclear reprogramming [35]. Since CB is transiently applied to SCNT mouse oocytes to prevent polar body extrusion upon activation, we cannot rule out a role for actin in the initial phase of reprogramming that precedes the first cell division.

Irrespective of which process – DNA replication or cytokinesis – was experimentally inhibited, the response was similar in SCNT and fertilized embryos. The similar response of SCNT and fertilized embryos to the block of the first round of DNA replication suggests that the requirement of DNA replication is not specific in regard to the oocyte manipulation procedure (SCNT vs ICSI). Either the molecular machinery involved in oocyte-mediated reprogramming shares components with the machinery responsible for processing the sperm nucleus, or Aph affects converging parts of distinct pathways. This is consistent with the hypothesis that mechanisms of reprogramming may be nothing but the same mechanisms in place for fertilization, hijacked to process somatic chromatin instead of sperm chromatin [36].

There is one final issue raised by our study. So far, problems with cloning have been blamed on the failure of gene expression reprogramming, and this is probably the main cause overall. However, even among the fertilized embryos, there was a proportion that did not contain detectable mRNA for Oct4 or Nanog, despite the presence of β-actin mRNA. In previous studies, fertilized mouse embryos without detectable Oct4 mRNA accrued 10% after IVF/ICSI, as compared to 0% or max. 3–4% for blastocysts that were produced by fertilization *in vivo* [6], [32], [37]. After SCNT, 25% of the blastocysts were reported by Bortvin and colleagues to lack detectable Oct4 mRNA [37], which matches the 36% of blastocysts with no detectable Oct4 mRNA observed in the present study. In the case of *Nanog*, 44% and 27% of all SCNT and ICSI embryos (pooled results) were Nanog mRNA-negative but β-actin-mRNA positive. Taken together, our data show that *in vitro* manipulation *per se* has an effect on gene expression regardless of somatic reprogramming. Therefore, as remarked by Solter [6], we should be apprehensive about possible consequences for the genome and for the phenotype in micromanipulations conducted on oocytes, beyond the case of *Oct4*.

## Conclusions

Our results demystify the portrayal of oocytes as special reprogrammers: DNA replication is an integral part of the oocyte’s reprogramming machinery, similar to what has been observed in transcription factor-induced pluripotency. Furthermore, our results challenge the view that reprogramming occurs within hours following nuclear transfer into mouse oocytes, as seen for SCNT into zygotes [2]; reprogramming at the *Oct4* and *Nanog* loci does not happen within hours nor even within days without DNA replication. It also is interesting to note that the recipient zygotes in Egli and colleagues’ study had undergone one round of DNA replication, since they were used in M-phase. Last but not least, the similar effects seen in SCNT and fertilized embryos may indicate that the mere micromanipulation and *in vitro* culture contribute at least in part to the defects of oocyte-mediated reprogramming.

## Materials and Methods

### Mice

Six to 8-week-old C3H/HeN mice were used as metaphase II (MII) oocyte donors. Ten week-old C57Bl/6J mice were used as cumulus cell and sperm donors. These mice were reared in house under specified pathogen-free conditions, were housed in groups of 5 in Type II L individually ventilated cages, and were fed on Harlan 2020SX diet. In order to detect reprogramming by means of *Oct4* promoter driven GFP, C57Bl/6J mice were replaced with OG2 mice of either sex that carry an *Oct4-GFP* transgene (JAX stock number 004654). Mice were superovulated by intraperitoneal injection of pregnant mare serum gonadotropin (PMSG; Intergonan, Intervet, 10 IU) and human chorionic gonadotropin (hCG; Ovogest, Intervet, 10 IU) 48 hours apart. Mice were sacrificed by cervical dislocation and metaphase II oocytes were collected from oviducts 14 hours post hCG. Except for the hormone injections used to collect the oocytes, no *in vivo* procedure was conducted on the mice, which were maintained and used for superovulation according to institutional guidelines and approval by the local ethics committee (Landesamt für Natur, Umwelt und Verbraucherschutz, NRW, Germany; permit number 87–51.04.2010.A387).

### SCNT (Somatic Cell Nuclear Transfer)

SCNT was performed as previously described [38]. Briefly, the chromosomal spindle of C3H/HeN oocytes was removed from recipient oocytes by gentle suction in a piezo-operated microcapillary needle (10 µm inner diameter, back-loaded with mercury) in the presence of cytochalasin B (CB, 1 µg/ml in Hepes-buffered CZB medium). A single C57Bl/6J or OG2 cumulus cell nucleus was transplanted into enucleated ooplasts by injection with a piezo-operated microcapillary needle (7 µm inner diameter, back-loaded with mercury) in the presence of polyvinylpyrrolidone (m.w. 40 kDa, 1% in Hepes-buffered CZB medium). The nucleus-transplanted ooplasts were parthenogenetically activated in Ca^2+^-free α-MEM supplemented with 10 mM SrCl_2_ and 5 µg/ml CB. All micromanipulations were conducted in Hepes-buffered CZB medium (containing 5.56 mM glucose) under Nomarski optics at 29°C room temperature. Recovery from micromanipulation was allowed in α-MEM medium (ooplasts) or in α-MEM and Hepes-buffered CZB medium mixed 1∶1 (nucleus-transplanted ooplasts) for 1 hour.

### Intracytoplasmic Sperm Injection (ICSI)

ICSI was performed by injecting a sperm head (C57Bl/6J or OG2 epididymal sperm) into a MII stage C3H/HeN oocyte, in Hepes-buffered CZB medium, using a piezo drill (PrimeTech). Two hours after injection, oocytes with a second polar body were retained for further culture.

### *In vitro* Culture of SCNT and ICSI Embryos

*In vitro* culture of SCNT and ICSI embryos was performed as previously described [39]. α-MEM was purchased (Sigma, M4525) and supplemented with BSA (2 mg/ml) and gentamicin sulphate (50 µg/ml). Embryos were cultured in groups of ∼100 in 500 µL of α-MEM medium in 4-well plates (Nunclon) without oil overlay in a humidified 37°C incubator with 5% CO_2_ in air.

### Pharmacological Inhibition of DNA Replication and Cytokinesis

Aphidicolin (Aph) (Calbiochem, 178273) was used at a concentration of 2 µg/ml to inhibit DNA synthesis. Cytochalasin B (CB) (Calbiochem, Cat. No. 250233) was used at a concentration of 5 µg/ml to inhibit network formation of actin filaments, thereby preventing cytokinesis. Stock solution of Aph and CB were prepared in dimethyl sulfoxide at 10 mg/ml and 5 mg/ml, respectively, stored at −80°C and diluted fresh to 2 µg/ml (Aph) and 5 µg/ml (CB) before each use.

### Assays for DNA and mRNA Synthesis

5-ethynyl-2′-deoxyuridine (EdU) and 5-ethynyl uridine (EU) were used to detect DNA and mRNA synthesis, respectively. For the incorporation of EdU during S-phase, the culture medium was supplemented with EdU (10 µM final concentration) prior to culture of the embryos with or without Aph for 30 min. For the incorporation of EU, a culture medium containing 1 mM EU was used to culture the embryos with or without Aph for 1 hour. Later on, according to the instructions, the embryos were fixed with 3.7% paraformaldehyde for 15 min followed by permeabilization by 0.5% Triton X-100 for 15 min at room temperature, then incubated with the Click-iT reaction cocktail for 30 min in the dark. The detection of incorporated EdU was performed using Click-iT EdU Alexa Fluor 647 imaging kit (Invitrogen, Cat. No. C10340) according to the manufacturer’s protocol. The detection of incorporated EU was performed using Click-iT RNA imaging kit (Invitrogen, Cat. No. C10330) according to the manufacturer’s instruction. Images were taken on a Nikon TE2000 microscope fitted with an UltraView RS3 confocal module.

### Transcriptome Analysis

Embryos from SCNT and fertilization with/without Aph treatment in 3 replicates, MII oocytes and cumulus cells were lysed and processed for transcriptome analysis by microarray using the Illumina BeadStation 500 (Illumina, San Diego, CA, USA) platform as previously described by Schwarzer and colleagues [40]. Briefly, total RNA was extracted using the ZR RNA MicroPrep kit (Zymo Research, R1061). Biotin-labeled cRNA was generated using the TargetAmp 2-Round Biotin-aRNA Amplification Kit 3.0 (Epicentre, TAB2R71024), of which 150 ng/µl was used for each hybridization reaction of 17 hours onto Mouse WG6 v2 expression BeadChips (Illumina). Chips were stained with streptavidin-Cy3 (GE Healthcare) and scanned using an iScan reader (Illumina). Thereafter, the bead intensities were mapped to gene information via BeadStudio 3.2 (Illumina). Microarray data of the embryos from SCNT and fertilization *in vitro* with/without Aph treatment, and from oocytes, were deposited at the NCBI Gene Expression Omnibus [41] and are accessible through GEO Series accession number GSE53497 (http://www.ncbi.nlm.nih.gov/geo/query/acc.cgi?token=gxihoikadlijfed&acc=GSE53497).

### Nested PCR Analysis of Oct4 and Nanog mRNA

RNA from single embryos was extracted using ZR RNA MicroPrep kit (Zymo Research, Cat. No. R1061). Complementary DNA synthesis was performed using the High Capacity cDNA Reverse Transcription Kit (Invitrogen) according to manufacturer’s instruction. A nested PCR was performed to amplify part of the *Oct4* cDNA containing a BamH1 sensitive single nucleotide polymorphism (SNP). Primer sequences of Oct4 were: Primer set 1: forward primer: GTCCCTAGGTGAGCCGTCTT; reverse primer: TCGAACCACATCCTTCTCTAGC with PCR conditions being 95°C 5 min; 95°C 30 s; 59°C 30 s; 72°C 50 s; 25 cycles; 72°C 5 min. Primer set 2: forward primer: GTGAGCCGTCTTTCCACCAG; reverse primer: AACACCTTTCCAAAGAGAACGC with PCR conditions being 95°C 5 min; 95°C 30 s; 59°C 30 s; 72°C 50 s; 32 cycles; 72°C 5 min. The PCR product of Oct4 was digested for 10 min at 37°C using FastDigest BamH1 (Thermo Scientific) followed by agarose gel electrophoresis. Primer sequences for Nanog’s nested PCR were: Primer set 1: forward primer: TACCTCAGCCTCCAGCAGAT; reverse primer: CCTCCAAATCACTGGCAG with PCR conditions being 95°C 5 min; 95°C 30 s; 59°C 30 s; 72°C 50 s; 25 cycles; 72°C 5 min. Primer set 2: forward primer: AACCTGAGCTATAAGCAGGTTAAGA; reverse primer: TTATGGAGCGGAGCAGCATT; with PCR conditions being 95°C 5 min; 95°C 30 s; 59°C 30 s; 72°C 50 s; 32 cycles; 72°C 5 min. The PCR product was analyzed by agarose gel electrophoresis. β-actin was amplified as a loading control and the same nested PCR conditions as for Oct4 and Nanog were used. The primer sequences for β-actin were: Primer set 1: forward primer: AGGTCATCACTATTGGCAAC; reverse primer: ACTCATCGTACTCCTGCTTG. Primer set 2: forward primer: TCCTTCTTGGGTATGGAATCCTGT; reverse primer: TGGCATAGAGGTCTTTACGGA.

### Live-cell Imaging of OCT4-GFP

Embryos produced by SCNT of OG2 cumulus cells or by ICSI with OG2 sperm were imaged on the stage of a Nikon TE 2000 inverted microscope fitted with a Nikon ACT2U camera system, using a 10× objective and a fixed exposure time of 1 second. A mercury bulb provided blue light excitation for GFP.

### Statistical Analysis

We applied t-test to compare intensity measurements of EdU and EU, and chi-square test to compare the proportions of embryos expressing Oct4 and Nanog transcripts. P<0.05 was considered statistically significant (considering the conservative Bonferroni correction for multiple comparisons). For gene ontology analysis, we used the **G**ene **O**ntology en**RI**chment ana**L**ysis and visua**L**iz**A**tion tool (GORILLA) applying the False Discovery Rate (FDR) with Benjamini-Hochberg correction for multiple comparisons to compare enrichment in the ‘biological process’.

## Supporting Information

Table S1 **List of genes whose mRNAs are upregulated (≥2-fold) at 96 hrs post-activation relative to 6 hrs post-activation in SCNT and ICSI embryos, with or without Aph treatment.** The mRNAs that are upregulated in both Aph^+^ and Aph^−^ specimenst are subjected to GO analysis.
(XLSX)
